# Malignant peripheral nerve sheath tumor of the breast: case report

**DOI:** 10.1186/1477-7819-5-142

**Published:** 2007-12-21

**Authors:** Kajal Kiran Dhingra, Shramana Mandal, Somak Roy, Nita Khurana

**Affiliations:** 1 Department of Pathology, Maulana Azad Medical College and associated Lok Nayak Hospital, Bahadur Shah Zafar Marg, New Delhi 110002, India

## Abstract

**Background:**

Malignant peripheral nerve sheath tumor is a rare soft tissue sarcoma of ectomesenchymal origin. It is the malignant counterpart of benign soft tissue tumors like neurofibromas and schwannomas and may often follow them. Common sites include deeper soft tissues, usually in the proximity of a nerve trunk. Breast is an extremely rare location of this lesion and presentation as a breast lump in the absence of pain or previous benign neural tumor is even rarer.

**Case presentation:**

A 38-year-old female presented with complaints of painless, hard breast lump for three months which was clinically suspected to be a ductal carcinoma with inconclusive fine needle aspiration cytology. Histopathology revealed a malignant spindle cell tumor which was confirmed to be malignant peripheral nerve sheath tumor on the basis of immunopositivity for vimentin, neurone specific enolase and S-100.

**Conclusion:**

To the best of our knowledge only six such case reports have been published in literature. The differential diagnosis of malignant peripheral nerve sheath tumor should be considered by the clinician as well as the pathologists in the work-up of a breast neoplasm as treatment and prognosis of this rare malignancy is different.

## Background

Malignant peripheral nerve sheath tumor (MPNST) is a rare soft tissue sarcoma of the ectomesenchymal origin. It is the malignant counterpart of benign soft tissue tumors like neurofibromas and schwannomas and may follow them. Common sites include deeper soft tissues, usually in the proximity of a nerve trunk. MPNST in the breast is often unsuspected and the diagnosis may be missed unless clinical suspicion is high and immunohistochemistry carried out

## Case presentation

A 38-year old female patient presented to the surgical out patient department with complaints of a rapidly growing right breast lump for three months. There was no history of prior breast mass, pain, trauma, bleeding, discharge, or family history of breast cancer. On examination there was a single 3.5 × 3 × 3 cm, ill defined non tender, firm, fixed mass in the right upper outer quadrant. There was no retraction or ulceration of the overlying skin. Fine needle aspiration cytology (FNAC) suggested a malignant spindle cell tumor probably a malignant phylloides. An excisional biopsy was advised to confirm the diagnosis and to guide the further management.

Grossly the specimen was a skin covered lumpectomy specimen measuring 5 × 4.4 × 4 cms. The cut sections revealed a firm infiltrative unencapsulated gray white tumor measuring 3 × 3 × 2.8 cms. There were no slit like areas, areas of hemorrhage, necrosis or calcification.

**Figure 1:**
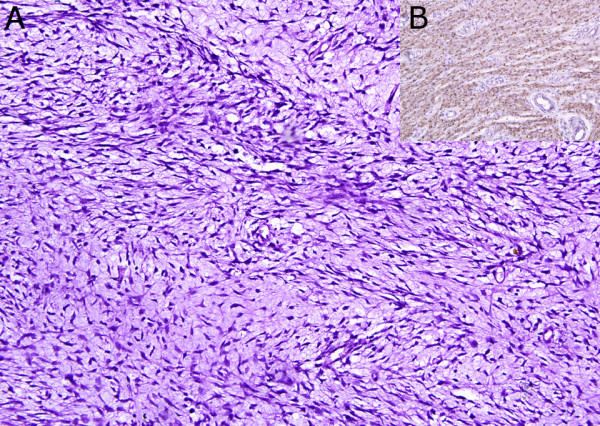
**a. **Alternating hyper and hypocellular areas of tumor with palisading spindle shaped cells and intervening myxoid areas (H&E ×200). Cells are diffusely positive for S-100 (**b**) (Immunoperoxidase ×200)

**Figure 2:**
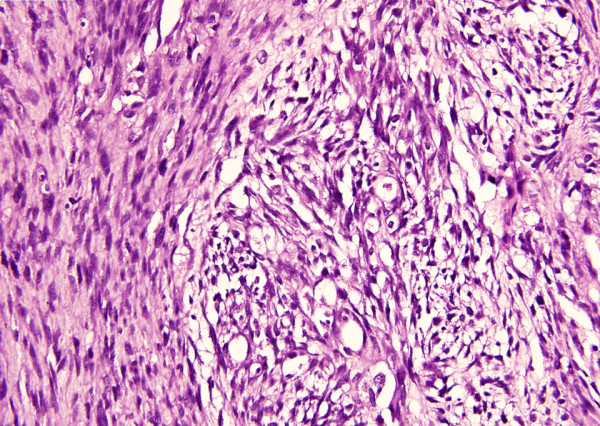
Hyperchromatic and pleomorphic wavy nuclei with mitoses. (H&E ×400)

Microscopically, Hematoxylin and Eosin (H&E) stained sections revealed a highly cellular tumor having intervening myxoid areas with streaming of spindle shaped cells highly suspicious of an MPNST (Figure 1a). The wavy nuclei and alternating hyper and hypo-cellular areas further suggested a tumor of neurogenic origin. Nuclei were hyperchromatic and pleomorphic with abundant mitoses. There were no entrapped epithelial elements (Figure 2). A provisional diagnosis of malignant spindle cell sarcoma was made. Immunohistochemistry was done to categorize the tumor. The tumor cells were also positive for neuron specific enolase (NSE) and Vimentin. S-100 was diffusely positive in the tumor cells confirming the neural nature of tumor and the final diagnosis of MPNST was made. (Figure 1b)

## Discussion

Malignant peripheral nerve sheath tumor (MPNST) is the preferred term for tumors originating from peripheral nerves or from cells associated with the nerve sheath, such as Schwann cells, perineural cells replacing previous entities such as malignant schwannoma, malignant neurilemmoma and neurofibrosarcoma. They represent approximately 10% of all soft tissue sarcomas [1]. MPNSTs commonly arise in adult patients ranging from 20 to 50 years of age. They originate from a major or minor peripheral nerve branch or its sheath. The common sites of origin include the extremities and trunk usually sciatic nerve, brachial plexus and the sacral plexus. Most cases average more than 5 cm in diameter [2]. They may arise spontaneously, although in 5% to 42% of cases an association with Neurofibromatosis (NF) Type 1 is known. Deeper neurofibromas are more likely to undergo malignant transformation rather their superficial counterparts in NF Type 1 [3]. MPNST of the breast is very rare and has been reported only twice in English literature and a total of six case reports in the extensively searched medical literature till date [4-9].

MPNST have to be distinguished from malignant phylloides, fibrosarcoma and leiomyosarcoma. Malignant phylloides shows features reminiscent of phylloides tumor, with compressed epithelium lined leaf like spaces, which were absent in the present case despite exhaustive sampling of the tumor. MPNST closely resembles fibrosarcoma morphologically. MPNST is comprised of spindle cells arranged in dense cellular areas interspersed with hypocellular myxoid areas and these cells show wavy nuclear contours which helps in excluding a fibrosarcoma. On the other hand, spindle cells of the leiomyosarcoma have a more distinct eosinophilic cytoplasm and a blunted nucleus compared to those of MPNST thus ruling out the diagnosis in this case. Distinguishing the MPNST from a benign nerve sheath tumor may be challenging too, as occasional neurofibromas may be quite cellular and contain occasional pleomorphic cells but presence of high mitotic activity was the determining feature in our case [1].

The MPNST is classified into three types – epithelioid, mesenchymal (including Triton tumor) and glandular variants. The epithelioid variant demonstrates plump, rounded cells scattered in lesion, usually in rather small numbers or in well defined clusters as seen in our case [10].

Immunostaining shows focal staining for S-100, CD57 and Leu-7 and myelin basic protein in 50% of the cases. The present case expressed diffuse and strong positivity for S100 confirming the diagnosis [11]. MPNST in most instances has a poor 5 year survival rate. Treatment is complete surgical excision of the tumor with negative margins along with radiotherapy for best outcome with respect to local recurrence and distant metastases. Dissection of axillary tail is not the protocol as the mode of dissemination is primarily hematogenous and not through the axillary lymph nodes [12].

## Conclusion

Our case of MPNST is unique because of its rare location. We report the seventh case of MPNST of the breast. Also the patient had no pain at the time of presentation and no history of long standing preceding neurofibromas was present. On extensive examination there were no subcutaneous swellings, *café au lait *spots or Lisch nodules ruling out underlying Neurofibromatosis.

## Abbreviations

FNAC – Fine Needle Aspiration Cytology

H&E – Hematoxylin & Eosin

MPNST – Malignant Peripheral Nerve Sheath Tumor

NF – Neurofibromatosis

## Competing interests

The author(s) declare that they have no competing interests.

## Authors' contributions

KKD, SM, SR and NK all contributed to the drafting of the manuscript.

KKD and SM proof read the documents thoroughly and helped in revising the manuscript. SR carried out the immunohistochemistry and digital microphotography and image editing and contributing to pathological aspects of the manuscript.

NK revised the final proof and photomicrographs thoroughly and gave final approval to the work. All authors read and approved final manuscript.
